# A Manual of Medicine

**Published:** 1903-03

**Authors:** 


**A Manual of Medicine.** Edited by W. H. ALLCHIN, M.D. Lond. Vol. IV. "Diseases of the Respiratory and of the Circulatory Systems." Pp. xi., 493. London: Macmillan and Co., Limited. 1902.

Dr. Mitchell Bruce writes on Diseases of the Heart and Blood-vessels; Dr. F. de Havilland Hall, on Diseases of the Pleura; Dr. Leonard Hill, on the Physiology of Respiration and the Physiology of the Circulation; Dr. Hector Mackenzie, on Diseases of the Lower Respiratory Tract; Dr. Lewis Smith, on Diseases of the Upper Respiratory Tract; and the Editor, on Disorders of the Diaphragm and Dropsy. The treatment of pneumonia is dismissed in two pages, and no mention is made of such important drugs as digitalis, phenacetine, chloral, and ether: the ice-bag is mentioned in a cursory way, but no details as to its use are given; this is the case also as regards pericarditis, in which condition we believe the ice-bag to be of the greatest value. The general excellence of the work has been commented on in our previous Review, vol. xix., p. 151, and this volume is full of interest and instruction for those who study it attentively. It is intended for the youthful student, as the type is small for those above middle-age.

